# Joint Report of One Hundred Observations Made with a View to the Determining of the Sex in Utero

**Published:** 1874-08-15

**Authors:** Albert B. Strong, D. A. K. Steele


### MEDICAL EXAMINER.

A

*Semi-Monthly Journal of Medical Sciences.*

EDITED BY N. S. DAVIS, M.D., AND F. H. DAVIS, M.D.

No. XVI. CHICAGO, AUG. 15, 1874. VOL. XV.

### Original Communications.

### JOINT REPORT OF ONE HUNDRED OBSERVATIONS MADE WITH A VIEW TO THE DETERMINING OF THE SEX IN UTERO.

BY ALBERT B. STRONG, M.D., AND D. A. K. STEELE, M.D.

*Read before the Chicago Medical Society, July* 20, 1874.

THE interesting fact that, in a certain proportion of cases, we may determine with some degree of accuracy the sex of the child in utero by auscultation, has long been established. It is claimed that the pulse of the female fœtus is uniformly much faster than that of the male, one observer establishing a difference between them of fifteen or twenty pulsations per minute. It is also claimed that the presentation may be actually determined by noting the point at which these sounds are most distinctly audible.

Some writers have been very enthusiastic in defence of the opinions founded upon their observations. Frankenhauser, for example, gravely asserts (*British Med. Jour.*, Aug. 6, '73), that in fifty cases which he examined, his diagnosis was in every instance correct. Other writers, more conscientious, perhaps, or less careful, have declared that their observations have proved comparatively worthless. In an article on Fœtal Physical Diagnosis, by Frank C. Wilson, M.D., visiting physician to the Louisville City Hospital (*Amer. Practitioner*, Dec., 1873), the writer says he has "kept accurate notes of all the cases met in hospital or private practice, diagnosing the sex in each, failing only in nine cases out of 109." The average pulse of the males was 125; of the females, 143; 134 being the average of both sexes and constituting a sort of dividing line between them. In Dr. Cumming's forty-one cases, the average pulse of the males was 131; of the females, 145; of both sexes, 136.

In reviewing the literature of this subject, which is exceedingly scanty, we have been much surprised to observe that conclusions similar to those given above are so radically different from our own. On the 1st of February last, we commenced a series of observations, in order to ascertain in what percentage of cases, predictions as to sex could be verified, and have each kept a careful record of fifty separate cases in which observations have been made since that date. Other interesting facts have been noted, which are also subjoined, as concisely as possible in the preparation of a detailed statement. From these it will appear that the disparity between the pulse of the male and the female infant is not as great as that established by other observers, and that, as a consequence, the ability to predict the sex of the fœtus in utero is far from flattering. The statistics we present are of such a character, that those disposed to do so may draw from them their own conclusions—a privilege we do not find accorded by those whose observations have been quoted. The weight of most of the children whose fœtal pulse was registered, was also determined, with a view to establishing a relation between the vigor of the individual and the rapidity of the pulse. But we do not find ourselves justified in establishing any connection between them.

The stethoscope was found preferable to the unaided human ear in noting the cardiac pulsations, since the latter are limited to a space but two or three square inches in extent. It should be added that, for convenience, we divided the uterine tumor, by four imaginary right lines, into four equal parts, designated as the right and left upper, and the right and left lower quarters. It is stated that if the heart pulsates in the lower half of the abdomen, the vertex presents; if in the upper, the breach is the presenting part; and, furthermore, that the occiput or coccyx will be on that side of the median line where the heart-sounds are most distinctly audible.

Two sounds can be heard in the abdomen of the pregnant female, apart from those which originate from the movement of the contents of the peristaltic intestine. These are the sound of the fœtal heart, and the placental souffle, which is synchronous with the maternal pulse. The latter is a loud blowing murmur, frequently of a musical character, and not at all constant, being plainly audible at one time and absent at another. It differs very materially from the heart-sounds, which are independent of the maternal circulation, and are short, sharp, clear and clicking, similar in character to the second sound of the adult heart. The fœtal pulse is subject to the same variations as that of the adult, only in a much more marked degree. While the child is perfectly quiet the heart is tolerably uniform in its pulsations, but by the least movement the pulse is greatly accelerated, increasing thirty to forty beats in the minute. The cause being removed, this increase in the frequency of the pulse rapidly disappears. It is said that the heart-sounds cannot always be heard in the living child. Such cases must be rare, especially in the last month of pregnancy, for we never have failed of detecting the pulse after a careful and, in some cases, prolonged examination. Frequently at the commencement of the observation the souffle has been so loud as to mask all other sounds, but as the momentary excitement of the mother passed away, the heart could be heard. Throughout a natural labor the action of the heart is variable, so much so that in the majority of cases when observation only was made at this time, the prognosis proved to be incorrect. During the first part of a pain the pulse is greatly accelerated, but as the uterus contracts more firmly and the child is subjected to greater pressure, it falls considerably; as the pain disappears it rises above the normal rate, and if the interval lasts a few moments, it again falls and becomes regular and more rapid than before labor began. For example, in one case the pulse was steady for several days at 124; during the first part of a pain it increased to 150; during the latter part it sank to sixty; during the first part of the interval it increased to 170, and then decreased and became steady at 134. From these facts relative to the variable action of the fœtal heart, it is evident that the examination *must be repeated at different intervals and during the time that the child is perfectly quiet;* and that the average result of such examination, since it is an approximation to the true state of the pulse, can alone be of possible service in determining the sex of the child.

Of the first fifty women examined, there were:

Multiparæ, 13; primiparæ, 37.

NATIONALITY OF MOTHER.

United States, 16; Sweden, 9; Ireland, 7; Germany, 6; England, 6; Norway, 4; Denmark, 1; Switzerland, 1. Foreign, 68 per cent. of the whole number, and native, 32 per cent.

AGE OF MOTHER.

February—Maximum, 37 years; minimum, 18 years; average, 25 years.

March—Maximum, 37 years; minimum, 17 years; average, 25 years.

April—Maximum, 40 years; minimum, 17 years; average, 24 years.

May—Maximum, 25 years; minimum, 16 years; average, 21 years.

June—Maximum, 32 years; minimum, 24 years; average, 27 years.

Grand total—Maximum, 40 years; minimum, 16 years; average, 24 years.

DURATION OF LABOR.

February—Maximum, 20 hours; minimum, 3 hours; average, 13 hours.

March—Maximum, 18 hours; minimum, 4 hours; average, 11 hours.

April—Maximum, 36 hours; minimum, 6 hours; average, 13 hours.

May—Maximum, 36 hours; minimum, 1 hour; average, 11 hours.

June—Maximum, 24 hours; minimum, 8 hours; average, 13 hours.

Grand total—Maximum, 36 hours; minimum, 1 hour; average, 12 hours.

WEIGHT OF CHILDREN.

February—Maximum, 10 pounds; minimum, 6 pounds; average, 8¼ pounds.

March—Maximum, 10 pounds; minimum, 8 pounds; average, 8¾ pounds.

April—Maximum, 10 pounds; minimum, 6 pounds; average, 8 pounds.

May—Maximum, 11¼ pounds; minimum, 4 pounds; average, 8 pounds.

June—Maximum, 9½ pounds; minimum, 5½ pounds; average, 8 pounds.

Grand total—Maximum, 11¼ pounds; minimum, 4 pounds; average, 8 1-5 pounds.

SEX OF CHILDREN.

February—Male, 5; female, 3.

March—Male, 10; female, 3.

April—Male, 8; female, 5.

May—Male, 10; female, 2.

June—Male, 4; female, 2.

Grand total, 50—Male, 35; female, 15.

RECORD OF FŒTAL HEART.

February—Average male, 138; average female, 137; average, 137½.

March—Average male, 128; average female, 139; average, 133½.

April—Average male, 131; average female, 140; average, 135½.

May—Average male, 127; average female, 125; average, 126.

June—Average male, 123; average female, 130; average, 126½.

Grand total—Average male, 129; average female, 136; average, 132.

PREDICTIONS.

Male—Correct, 24; doubtful, 4; incorrect, 7.

Female—Correct, 8; doubtful, 3; incorrect, 4.

STATISTICS FROM OBSERVATION OF SECOND FIFTY CASES.

| No. Patients. | Date of Observation. | Pulsation of heart. | Where heard. | Date of Delivery. | Presentation. | Position. | Sex. | Weight, lbs. |
|---|---|---|---|---|---|---|---|---|
| 1 | Sept. 18, 1873, | 124 | Left lower quarter | Sept. 30th, | Ver. | 1st | | |
| 1 | 2nd stage of labor | 132 | " | | | | | |
| 2 | Sept. 16th, | 128 | " | Oct. 1st, | Ver. | 1st | F | |
| 3 | " " | 132 | Right lower quarter | " 4th, | Ver. | 2nd | F | |
| 3 | " 22nd, | 140 | " | | | | | |
| 3 | Oct. 1st, | 140 | " | | | | | |
| 4 | " " | 140 | " | " 1st, | Ver. | 2nd | M | |
| 5 | " 11th, | 128 | Left lower quarter | " 11th, | Ver. | 2nd | M | 9 |
| 6 | " 6th, | 124 | Right lower quarter | " 11th, | Ver. | 1st | F | 7½ |
| 7 | " 8th, | 130 | Left lower quarter | " 16th, | Ver. | 1st | F | 8½ |
| 7 | 1st stage of labor | 140 | | | | | | |
| 7 | 2nd " " " | 144 | | | | | | |
| 8 | Oct. 9th, | 154 | " | " 16th, | Ver. | 1st | F | 9 |
| 9 | 2nd stage of labor | 140 to 160 | Median line, between pubes and umbilicus | " 25th, | Br. | 1st | M | 5½ |
| 10 | Oct. 10th, | 130 | Left lower quarter | Nov. 1st, | Ver. | 2nd | F | 8 |
| 10 | 1st stage of labor | 142 | | | | | | |
| 11 | Oct. 6th, | 128 | Right lower quarter | Nov. 1st, | Ver. | 2nd | F | 8¼ |
| 11 | " 10th, | 128 to 140 | " | | | | | |
| 11 | " 27th, | 120 | | | | | | |
| 11 | 2nd stage of labor | 130 | | | | | | |
| 12 | Oct. 8th, | 128 | Left lower quarter | Nov. 1st, | Ver. | 2nd | M | 11 |
| 12 | " 22nd, | 132 | | | | | | |
| 13 | Oct. 31st, | 124 | Median line, between pubes and umbilicus | Oct. 31st, | Ver. | 1st | M | 9 |
| 14 | " 28th, | 132 | All over abdomen | Oct. 31st, | Ver. | 1st | M | 8 |
| 15 | Nov. 4th, | 122 | Left lower quarter | Nov. 9th, | Ver. | 1st | F | 7¼ |
| 15 | 1st stage of labor | 144 | | | | | | |
| 16 | Oct. 20th, | 134 to 145 | " | Nov. 4th, | Ver. | 1st | F | 7 |
| 17 | " 20th, | 118 to 140 | Left lower quarter | Nov. 9th, | Ver. | 2nd | F | 8 |
| 17 | " 22nd, | 220 | | | | | | |
| 18 | Nov. 10th, | 132 | " | " 11th, | Ver. | 1st | F | 6 |
| 19 | Oct. 2nd, | 140 | " | " 13th, | Ver. | 1st | F | 7½ |
| 19 | Nov. 6th, | 134 | | | | | | |
| 20 | " 2nd, | 132 | Right lower quarter | Nov. 13th, | Ver. | 2nd | F | 7 |
| 21 | Oct. 28th, | 130 | Left lower quarter | " 13th, | Ver. | 2nd | M | 7¼ |
| 21 | Nov. 2nd, | 144 | | | | | | |
| 22 | " 16th, | 130 | " | " 16th, | Ver. | 1st | M | 8½ |
| 23 | Oct. 31st, | 122 | " | " 18th, | Ver. | 2nd | M | 8 |
| 23 | Nov. 3d, | 132 | | | | | | |
| 24 | Oct. 10th, | 130 | Right lower quarter | Nov. 23d, | Ver. | 2nd | M | 7 |
| 24 | " 13th, | 140 | | | | | | |
| 24 | Nov. 4th, | 128 to 140 | Over most all of the uterine tumor | | | | | |
| 24 | " 15th, | 126 to 140 | Right side | | | | | |
| 25 | Oct. 9th, | 150 | Left lower quarter | Nov. 25th, | Ver. | 1st | M | 10 |
| 25 | " 13th, | 140 | | | | | | |
| 25 | " 22nd, | 140 | | | | | | |
| 25 | Nov. 15th, | 134 | | | | | | |
| 26 | Nov. 10th, | 124 | " | Nov. 27th, | Ver. | 1st | M | 8¼ |

STATISTICS FROM OBSERVATION OF SECOND FIFTY CASES—Continued.

| No. Patients. | Date of Observation. | Pulsation of heart. | Where heard. | Date of Delivery. | Presentation. | Position. | Sex. | Weight, lbs. |
|---|---|---|---|---|---|---|---|---|
| 26 | Nov. 23d, | 118 | | | | | | |
| 26 | " 25th, | 124 | | | | | | |
| 27 | " 25th, | 150 | Left lower quarter | Nov. 26th, | Ver. | 1st | F | 6 |
| 28 | Oct. 27th, | 130 to 180 | Over two-thirds of tumor | Nov. 30th, | Ver. | 2nd | M | 9½ |
| 28 | Nov. 23d, | 128 to 140 | Most intense in right lower quarter | | | | | |
| 29 | Nov. 30th, | 132 | Right lower quarter | Nov. 30th, | Ver. | 2nd | F | 7 |
| 29 | 2nd stage of labor | 146 | | | | | | |
| 30 | Nov. 10th, | 120 | " | Dec. 3d, | Ver. | 2nd | M | 8 |
| 30 | " 15th, | 140 | | | | | | |
| 30 | " 23d, | 140 | | | | | | |
| 31 | Oct. 2nd, | 152 | " | Dec. 5th, | Ver. | 2nd | M | 9 |
| 31 | " 15th, | 140 | | | | | | |
| 31 | " 23d, | 148 | | | | | | |
| 32 | " 31st, | 140 | Left lower quarter | Dec. 8th, | Ver. | 1st | M | 8 |
| 32 | Nov 18th, | 140 | | | | | | |
| 33 | " 8th, | 124 | " | Dec. 23d, | Ver. | 1st | M | 12 |
| 33 | " 15th, | 128 to 160 | | | | | | |
| 33 | " 23d, | 132 | | | | | | |
| 34 | " 26th, | 140 | | Dec. 25th, | Ver. | 1st | F | 6 |
| 34 | Dec. 1st, | 124 to 130 | | | | | | |
| 35 | " 28th, | 140 to 160 | " | Dec. 31st, | Ver. | 1st | F | 7 |
| 35 | " 30th, | 130 | | | | | | |
| 36 | " 26th, | 130 | " | Dec. 30th, | Ver. | 1st | F | 9 |
| 37 | " 31st, | 150 to 180 | Right lower quarter | Jan. 3d, | Ver. | 2nd | M | 8 |
| 38 | " 26th, | 134 | All over tumor | Jan. 11th, | Ver. | 1st | F | 8¼ |
| 39 | " 28th, | 130 | Left lower quarter | Jan. 7th, | Ver. | 1st | F | 7 |
| 39 | " 31st, | 138 | | | | | | |
| 40 | " 5th, | 132 | Right lower quarter | Jan. 9th, | Ver. | 2nd | F | 8½ |
| 40 | " 11th, | 130 | | | | | | |
| 41 | " 11th, | 132 | Left lower quarter | Jan. 1st, | Ver. | 1st | F | 7 |
| 42 | Jan. 11th, 1874, | 126 | Right lower quarter | Jan. 12th, | Ver. | 2nd | F | 7 |
| 43 | Dec. 26th, | 140 | Left lower quarter | Jan. 13th, | Ver. | 1st | F | 8 |
| 43 | " 31st, | 164 | | | | | | |
| 44 | Jan. 27th, 1874, | 170 | " | Jan. 28th, | Ver. | 1st | F | 6¼ |
| 45 | Nov. 23d, | 122 to 180 | Right lower quarter | Jan. 16th, | Ver. | 2nd | F | 11¼ |
| 45 | Dec. 11th, | 128 | | | | | | |
| 46 | Feb. 8th, 1874, | 128 | " | Feb. 8th, | Ver. | 1st | M | |
| 47 | Jan. 8th, | 150 | Left lower quarter | Feb. 9th, | Ver. | 1st | F | |
| 48 | " 16th, | 164 | " | Feb. 13th, | Ver. | 1st | M | |
| 49 | " 3d, | 130 | " | Feb. 14th, | Ver. | 1st | M | |
| 50 | " 12th, | 130 | Right lower quarter | Feb. 15th, | Ver. | 1st | M | |

From the foregoing statistics we deduce the following observations: 1. In the majority of cases male fœtal hearts are slower than female. 2. 132 fœtal pulsations per minute is the average which constitutes a dividing line between the sexes. Below this, sixty-eight and four-sevenths per cent. are males, twenty per cent. are females, eleven and three-sevenths per cent. are doubtful. Above this fifty-three and one-third per cent. are females, twenty-six and two-thirds per cent. are males, twenty per cent. doubtful. We have here, it may be observed, another demonstration of the fickleness of the female heart. 3. The most accurate observations are made during the last four weeks of gestation. 4. The rapidity of the heart's action is increased in proportion to the feebleness of the fœtus. 5. Calcareous or fatty degeneration of the placenta renders the pulsations feeble and irregular. 6. In some cases it would be possible to diagnose diseased conditions of the placenta from careful observation of the fœtal heart.

Of fifty cases examined consecutively twenty-seven gave birth to female children and twenty-three to males. The lowest rate observed was 118; it occurred but twice; once each in a male and female child. The highest rate noted was 180, occurring three times, twice in males and once in the case of a female. The average rate of the male pulse was 136.3; of the female, 137; of both sexes, 136.7. Considering the latter as the dividing line between both sexes, a pulse at and below this rate may be referred to males, and that above it to females. In twenty-six cases the sex was correctly predicted, and in twenty-four an error was made.

If the cases be excluded where there was unusual activity of the fœtus, the average rate of the male pulse will be found to be 133.6; of the female, 136.2; of both sexes, 134.7.

Considering, then, 134 as the dividing line between the sexes, the diagnosis was correct in twenty-four cases and incorrect in twenty-two. If, however, 128 be taken as the dividing line, the diagnosis was correct in twenty-eight cases and incorrect in twenty-two. There were six female children whose pulse was steady below 128; five males had a steady pulse between 128 and 138; three, between 138 and 148; two, between 148 and 158, and one between 158 and 168. So far as the facts elicited from these observations are of value, it is evident that they have utterly failed to furnish a basis for determining the sex in utero. Dr. Wilson admits the necessity of repeated examinations in each case, but says many of his observations were made but once. Possibly, if we had made repeated examinations to a greater extent, the result might have been different. In regard to determining the presentation, we can report more favorably, having been correct in forty-nine out of fifty cases. The case of failure was one where the breech was the presenting part, but the examination was not made till the second stage of labor. The determination of a position on the right or left of the median line, was correct in thirty-eight and incorrect in eight cases; of the four remaining cases, the heart-sounds were heard all over the uterine tumor in two, both vertex presentations, with the occiput to the left; in the other two cases the heart-sounds were the most distinct in the median line, between the pubes and umbilicus, one being a vertex presentation, with the occiput to the left, and the other a breech.

Obscuration of the fœtal sounds may result from thick abdominal walls, tense abdominal muscles, or the presence of excessive liquor amnii. The placental attachment is of course indicated by the characteristic souffle. It is unnecessary to again refer to the necessity of repeated examinations in order to determine these facts, as the most accurate predictions were based on the results of numerous observations, when the temporary excitement induced by a single examination was eliminated from the record.

Our observations were conducted by the aid of an ordinary Camman's stethoscope, and our experience has made it clear that more distinct sounds are audible when the bell of the instrument is moistened and applied to the abdomen without pressure, as the peculiar thrill of the fœtal heart is lost when the stethoscope is grasped by the fingers.

As regards the artificial divisions of the uterine tumor given above, we found the fœtal pulsations most distinct in the left lower quarter in thirty cases; in the left upper quarter in five, and in the right upper quarter in three, the latter all cases of breech presentation. All the others were vertex, forty-one in the first position, and six in the third. If the fœtal heart be heard most distinctly above the imaginary transverse line, the presentation is most likely that of a breech; if below the line, it is almost certainly a case of vertex presentation. Inspection and palpation, however, afford valuable information in the determination of the fœtal position, and exclusive reliance should not be placed upon auscultation.

In conclusion, it may be generally stated that we find an opinion as to the sex of the child, founded on the rate of the fœtal pulse, to be of little more value than a guess, while the presentation, generally, and the exact position, possibly, may be accurately determined.

